# Studies on mercury occurrence in inorganic constituents of Polish coking coals

**DOI:** 10.1007/s11356-018-1667-1

**Published:** 2018-03-12

**Authors:** Tadeusz Dziok, Andrzej Strugała, Adam Włodek

**Affiliations:** 1 Faculty of Energy and Fuels, AGH University of Science and Technology, Al. A. Mickiewicza 30, 30-059 Krakow, Poland; 2 Faculty of Geology, Geophysics and Environmental Protection, AGH University of Science and Technology, Al. A. Mickiewicza 30, 30-059 Krakow, Poland

**Keywords:** Coking coal, Inorganic constituents, Mercury, EPMA, Modes of occurrence

## Abstract

During the cokemaking process, a significant amount of mercury occurring in a coal blend is released to the atmosphere. One of the ways of reducing this emission is to reduce mercury content in a coal blend. This could be obtained through the coal washing process. The optimization of this process requires the knowledge of mercury occurrence in coal, especially in its inorganic constituents. A qualitative analysis of mercury occurrence in the inorganic constituents of Polish coking coals was performed using an electron probe microanalyzer (EPMA). For that purpose, selected samples of rejects and middling products derived from the washing process in dense media separators and jig concentrators were examined. The obtained results have confirmed a strong connection between mercury occurrence and the presence of sulfides (pyrite, marcasite, and chalcopyrite) in Polish coking coals. Significant amounts of mercury were also noticed for barite, siderite, and aluminosilicates. The highest value of mercury content, at the level of 0.100%, was obtained for marcasite. For the analyzed coals, the effectiveness of mercury removal in the washing process was determined by the forms of pyrite occurring in coal. The highest values of effectiveness of mercury removal were obtained in the case of coals for which the large framboidal pyrite aggregates with chalcopyrite overgrowths were noticed. It was also found that middling products were characterized by the occurrence of the Hg-rich overgrowths of pyrite on organic matter. To achieve a significant reduction in mercury content in clean coal, it is necessary to develop an effective method of removing this form of pyrite from hard coal.

## Introduction

Mercury is characterized by highly toxic properties (Li and Tse 2015). According to Pirrone et al. (2010), 30% of mercury emission to the environment is caused by human activities, and coal utilization processes constitute one of the main sources of its emission (Pacyna et al. 2016). This issue is really important for such countries as Poland and China, because their energy production sector is based on coal (Burmistrz et al. 2016; Wang et al. 2011). The annual mercury emission in Poland in 2014 was estimated at the level of 9.6 Mg (KOBiZE 2016). The proportion of mercury emissions from the coal-related sectors was as follows: combustion 93.4%, pig iron and steel production 4.6%, and coke production 0.3%.

The issue of mercury emission is very important for coke industry. The annual production of coke worldwide exceeds 700 million Mg (Mysiak and Jarno 2016). In the coal coking process, a significant amount of mercury occurring in a coal blend is released to the atmosphere (ACAP 2005). This emission occurs during the charging and pushing operations as well as in leaks on the battery (US EPA 1997b). Mercury emission factors from coking plants range from 0.01 to 0.038 g/Mg of coke produced (ACAP 2005; Konieczyński et al. 2012; US EPA 1997a). A certain amount of mercury remains in coke and the rest passes into raw coke gas and then to coke gas cleaning products. Coke is commonly used in the processes of pig iron and steel production and, thus, contributes to mercury emission from this industry sector (Wang et al. 2016). It should be also mentioned that the use of sorbents (Lopez-Anton et al. 2016; Yu et al. 2016) in order to reduce the mercury emissions from coking plants has a limited possibility of application.

One of the ways of reducing mercury emission from coking plants as well as of lowering mercury content in coal coking products is to reduce mercury content in the coal blend. This could be obtained in the coal washing process, which is well known and commercialized (Rallo et al. 2012). The possibility of removing significant amounts of mercury from coal in this process was confirmed in the following studies: Dziok et al. (2015b), Zajusz-Zubek and Konieczyński (2014), Pan et al. (2017), and Pyka and Wierzchowski (2016). However, the effectiveness of mercury removal from coal is varied and it is closely related to the mode of mercury occurrence in coal. Therefore, the optimization of mercury removal from coal in the process of coal washing requires the knowledge of mercury occurrence.

Generally, mercury in coal can be present both in its organic matter and its inorganic constituents. Mercury in organic matter is connected with sulfur (Dziok et al. 2015a; Diehl et al. 2004). Mercury in mineral matter is mainly associated with sulfides (pyrite, marcasite, cinnabar) and also with such inorganic constituents as Pb and Se minerals, clausthalite, chlorite, silicates, tiemannite, kleinite, getchellite, and Hg-rich gold (Diehl et al. 2004; Hower et al. 2008; Kolker 2012; Zhang et al. 2002). Moreover, mercury can be associated with carbonates (Zheng et al. 2008). The occurrence of various mercury compounds in coal is also suggested by the results presented in the works (Guo et al. 2017; Uruski et al. 2015). It is generally believed that mercury removal efficiency in the process of coal washing is determined by the type of pyrite occurring in coal (Diehl et al. 2004; Mastalerz and Drobniak 2005; Toole-O’Neil et al. 1999).

For Polish coking coals, the modes of mercury occurrence have not been sufficiently investigated so far. The results of our previous works (Dziok et al. 2015a, 2015b) suggest that the occurrence of mercury in coal is connected with the occurrence of sulfur, especially pyritic sulfur. However, such a hypothesis requires empirical confirmation with the use of adequate analytical methods. The aim of the presented examinations was to determine the mode of mercury occurrence in inorganic constituents of Polish coking coals. For those purposes, the qualitative analysis using the Super Probe Electron Probe Microanalyzer (EPMA) JXA-8230 was performed.

## Experimental and analytical procedures

### Analyzed samples

**Fig. 1:**
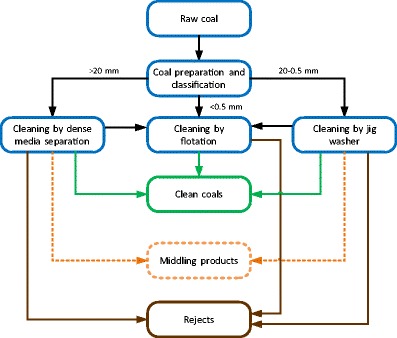
Basic scheme of coking coal preparation plant in Poland

In order to determine the amount of mercury removed from coking coal in the washing process, samples of raw coals and rejects were examined. The samples were obtained from the preparation plants of four Polish coking coal mines. A basic scheme of a coking coal preparation plant in Poland is shown in Fig. 1. Mercury content in analyzed samples was determined with the use of the MA-2000 Analyzer (Nippon Instruments Corporation) based on cold vapor atomic absorption spectroscopy (CVAAS) (Okońska et al. 2013). The obtained results are given in Table 1.

**Table 1:** Mercury content in analyzed samples of raw coals and rejects

Coal mine	Hg_t_^d^ [μg/kg]		Yield of rejects [%]
	Raw coal	Rejects	
B	86	85	42.2
C	60	59	29.1
E	117	180	54.3
F	98	108	46.3

In order to determine the effectiveness of mercury removal from coal in the washing process, the yields of rejects were estimated. The calculations were made based on the data on the mass of rejects generated in each of the analyzed processing plants in relation to the mass of raw coal subjected to the washing process. The data was obtained by the authors from the coal mine managements.

In order to identify the occurrence of mercury in the inorganic constituents of Polish coking coals, selected samples of rejects and middling products derived from the washing operation in dense media separators and jig concentrators were examined. Samples for the examinations were selected following the criterion of a different mode of occurrence of mercury in raw coal, i.e., coal subjected to the washing process. The selection was made based on the results of our previous work (Dziok et al. 2015a). In the cited work, the object of research consisted of different populations of samples obtained from six coking coal processing plants. In all the samples, the contents of ash, mercury, and total sulfur as well as pyritic, sulfate, and organic sulfur were determined. Next, a statistical analysis of the relationship between mercury content and the content of ash and the various forms of sulfur was performed. Based on the results of this analysis, conclusions concerning different ways of mercury association with sulfur in coal derived from each of the mines were formulated. Samples of rejects were selected from coal mines, for which the statistical analysis suggested a different mode of mercury occurrence in coal, i.e., its association with pyrite, other inorganic constituents, or various forms of sulfur found in coal. Additionally, selected samples of middling products derived from the coking coal washing operations were studied, for which the determined mercury contents were significantly higher than for clean coal and rejects produced in this process. The specification of the analyzed samples is given in Table 2 along with the justification of sample selection. The characteristics of the examined samples are shown in Table 3. The contents of moisture, ash, and total sulfur as well as pyritic, sulfate, and organic sulfur were determined according to ISO standards.

**Table 2:** Specification of analyzed samples

Sample no.	Coalmine	Justification of sample selection	Type of sample
I	B	Results of a statistical analysis suggest the association of mercury with various forms of sulfur found in coal (Dziok et al. 2015a)	Rejects derived from coal preparation plants
II	C	Results of a statistical analysis suggest the occurrence of mercury in inorganic constituents of coal other than pyrite (Dziok et al. 2015a)	
III			
IV	E	Results of statistical analysis suggest the occurrence of mercury in coal in pyrite (Dziok et al. 2015a)	
V	F		
VI	B	A significantly higher mercury content when compared to clean coal and rejects produced in the same process	Middling products derived from coal preparation plants
VII	E

**Table 3:** Characteristics of examined samples of rejects

Sample no.	*M*^ad^ [%]	*A*^d^ [%]	*Hg*_t_^d^ [μg/kg]	*S*_t_^d^ [%]	*S*_p_^d^ [%]	*S*_SO4_^d^ [%]	*S*_o_^d^ [%]
I	1.2	88.3	79	0.18	0.17	0.01	0.00
II	0.9	83.8	62	0.12	0.10	0.02	0.00
III	0.9	86.1	55	0.13	0.11	0.02	0.00
IV	1.8	77.8	249	0.95	0.87	0.08	0.00
V	0.8	79.7	114	0.29	0.26	0.02	0.01
VI	1.3	52.9	313	1.63	1.47	0.05	0,11
VII	1.6	33.8	246	0.62	0.29	0.01	0.40

*ad* air dried basis, *d* dry basis, *t* total, *p* pyritic, *SO4* sulfate, *o* organic

### Elemental composition analysis of examined samples using an electron probe microanalyzer

Chemical compositions of the analyzed phases were determined by the Electron Probe Microanalyzer (EPMA) JEOL JXA-8230 located at the Laboratory of Critical Elements KGHM – AGH. The JXA-8230 microanlyzer is characterized by a higher detection sensitivity for trace elements (JOEL 2017).

Based on stoichiometry, the identification of different minerals was performed. Simplified mineral formulas were calculated from the chemical composition obtained with the use of an EPMA. The [CO_3_]^2−^ content in carbonates was calculated as the complement to 100 wt%. Only in the case of pyrite/marcasite, the authors used the morphological criterion, assuming that pyrite forms regular, cubic crystals or framboids and marcasite forms globular or spherical aggregates. The selection of analytical points for the EPMA measurements was based on observations of the high-contrast backscattered electron images (BSE). Single-point measurements were chosen for most of the small, homogenous grains and multiple-point measurements were chosen only for large, inhomogeneous grains or zoned crystals.

In the measurements, the following conditions were used: the acceleration voltage of 20 kV (for sulfides) and 15 kV (for aluminosilicate minerals, oxides, and carbonates), the probe current of 20 nA, and the spot size of 1−5 μm. The following analytical lines, crystals, and standards were used for the measurements of sulfide minerals: S (Kα, PET, pyrite), Mn (Kα, LIF, alabandite), Fe (Kα, LIF, pyrite), Co (Kα, LIF, metallic Co), Ni (Kα, LIF, metallic Ni), Cu (Kα, LIF, metallic Cu), Zn (Kα, LIF, sphalerite), As (Lα, TAP, InAs), Se (Lα, TAP, metallic Se), Ag (Lα, PET, metallic Ag), Cd (Lα, PET, greenockite), In (Lα, PET, InAs), Sn (Lα, PET, SnS), Te (Lα, PET, Bi_2_Te_3_), and Hg (Mα, PET, HgTe).

Measurements of aluminosilicate minerals, oxides, and carbonates were performed, with the use of the following analytical lines, crystals, and standards: F - (Kα, LDE, fluorite). Na (Kα, TAP, albite). Mg (Kα, TAP, forsterite). Al (Kα, TAP, kyanite). Si (Kα, TAP, albite), P (Kα, PET, YPO_4_), Cl (Kα, PET, halite), K (Kα, PET, orthoclase), Ca (Kα, PET, wollastonite), Ti (Kα, LIF, rutile), Mn (Kα, LIF, metallic Mn), Fe (Kα, LIF, hematite), Zn (Kα, LIF, sphalerite), and Hg (Mα, PET, HgTe).

Measurements of trace elements (especially Hg) were carried out with the use of the L-type X-ray spectrometer (PETL, LIFL) and the H-type X-ray spectrometer (PETH, LIFH, TAPH). The L-type one is a large-crystal wavelength spectrometer with the 140-mm Rowland circle equipped with PET (Pentaerythritol) or LIF (lithium fluoride) crystals. The spectrometer of the H-type is a high-intensity wavelength spectrometer with the 100-mm Rowland circle equipped with PET, LIF, or TAP (thallium acid phthalate) crystals. Both types of spectrometers provide higher detection sensitivities than traditional spectrometers (JEOL 2012; Larnould 2013). Counting time and beam size were specified on the basis of mineral stability during the sample–beam interaction. Peak counting time was determined at 20 s for sulfide minerals (S) and 10 s for rock-forming minerals (R-F) while background time was determined at 10 s (S) and 5 s (R-F). The measurement time of mercury has been extended up to 30 s for peak counting time and 10 s for background time, to achieve lower detection limits and higher background to peak (B/P) ratios.

Hg Mα X-ray line was analyzed using a PET crystal mounted in an L-type spectrometer (*E* = 2.195 keV, PET L-position = 180.872) which is characterized by a low background level and high peak to background ratios. The nearest characteristic X-ray lines were Bi M_3_-N_1_ (I-order., PETL L-value = 177.335), Cu Kβ_1_, Kβ_3_ (IV-order., PETL L-value = 178.397), Pb M_3_-N_1_ (I-order., PETL L-value = 182.679), Bi Lα (V-order., PETL L-value = 183.202), Mn Kβ_3_, Kβ_1_ (III-order. PETL L-value = 183.572), and Zn Kα_1_ (IV-order. PETL L-value = 183.900. Possible interferences with enlisted X-ray lines were rejected due to their negligible intensities (high-order lines: Cu Kβ_1_, Kβ_3_, Bi Lα, Mn Kβ_3_, Kβ_1_, Zn Kα_1_, and very low contents of Bi and Pb in the analyzed phases). In the measurements of pyrite, marcasite, and chalcopyrite, negligible contents of Bi and Pb should not affect the Hg Mα measurements.

The lower limit of detection (LOD) for mercury measurements reached approximately 0.006% (60,000 μg/kg). Therefore, the EMPA method has allowed for the identification (the qualitative analysis) of inorganic constituents characterized by a relatively high mercury content, significantly higher than the total mercury content in the analyzed samples. For the obtained results of mercury content measurements, the expanded uncertainty at the confidence level of 95% was determined. The extended uncertainty varied from 0.005 to 0.009% with the average at the level of 0.007%. The values of the extended uncertainty determined for each case are presented in Figs. 9, 10, and 14 in the form of whiskers.

## Results and discussion

### Mercury emission from coking plant

**Fig. 2:**
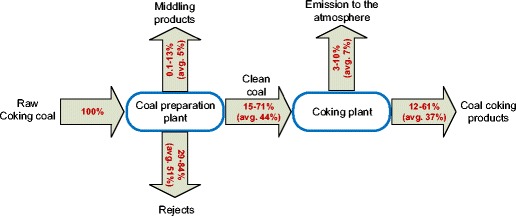
Distribution of mercury in coal washing and coking processes (converted to 1 kg of mercury contained in raw coal fed to coal preparation plant)

**Fig. 3:**
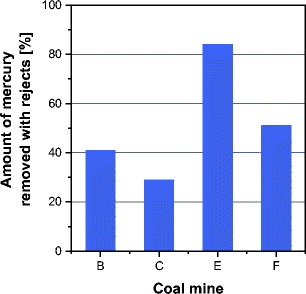
Comparison of values of effectiveness factors of mercury removal from coking coal in coal washing process

In Fig. 2, the distribution of mercury in the coal washing process and the coking process is presented. In the coal washing process, the gangue (in the form of rejects) is removed from raw coal. The gangue contains a number of impurities, including mercury. The amount of mercury removed from coking coal with rejects for the analyzed coal mines ranged from 29 to 84%, with the average value of 51% (Fig. 3). The effectiveness of mercury removal was estimated in accordance with formula (1). The effectiveness of mercury removal from coal is determined by both coal properties (Diehl et al. 2004; Mastalerz and Drobniak 2005) and the technological solutions of the washing process (Dziok et al. 2014). In the case of the analyzed coals, both the technological system of the washing process and their operating parameters did not differ significantly. Thus, in the authors’ opinion, the differences in mercury removal effectiveness should be explained by differences in coal properties, i.e., the washability of coal and the mode of mercury occurrence. The various modes of mercury occurrence in the analyzed coal were noticed in our previous work (Dziok et al. 2015a).

1 $$ {\eta}_{{\mathrm{Hg}}_{\mathrm{rejects}}}=\frac{\gamma_{\mathrm{rejects}}\times {\mathrm{Hg}}_{\mathrm{rejects}}}{{\mathrm{Hg}}_{\mathrm{rawcoal}}}, $$

where:

*η*_Hg_rejects_: the effectiveness factor of mercury removal from coal with rejects [%]
Hg_rejects_: mercury content in rejects [μg/kg]
Hg_raw coal_: mercury content in raw coal [μg/kg]
*γ*_rejects_: yield of rejects [%]

Clean coals derived from the coal washing process are used for coke production in coke ovens. The coking process is conducted in temperatures above 1000 °C. At such a high temperature, changes in the mineral matter of coal occur (Strugała 1998). These changes facilitate the release of mercury from coal and only a small amount of it remains in coke. A part of the released mercury passes into the atmosphere in the form of the fugitive emissions, occurring during the charging and pushing operations, as well as from leaks on the battery (US EPA 1997b). According to the available data for Polish coking plants, about 13.9% of mercury contained in clean coals is emitted to the atmosphere (Burmistrz et al. 2018). This corresponds to the reported emission factors (ACAP 2005; Konieczyński et al. 2012; US EPA 1997a). Due to the different mercury removal effectiveness for each of the analyzed coals in the washing process (from 29 to 84%), different values of emission factors in the coking process were obtained. Therefore, in accordance with formula (2), from 3 to 10% of mercury contained in raw coal is released into the atmosphere from the coking process. The rest of the mercury passes into the coking products (coke, benzole, tar, and sulfur) and, thus, may cause mercury emission to the atmosphere as a result of the further use of these products.

2 $$ {\mathrm{EF}}_{\mathrm{raw}}={\mathrm{EF}}_{\mathrm{cc}}\times \frac{\%{\mathrm{Hg}}_{\mathrm{cc}}}{100}, $$

where:

EF_raw_: mercury emission from coking plant in relation to mercury content in raw coal [%]
EF_cc_: mercury emission from coking plant in relation to mercury content in clean coal used for coke production [%]
%Hg_cc_: proportion of mercury passing into clean coal in washing process [%]

In comparison to the coal-based power generation sector, the reduction of mercury emission in the coke industry is much more difficult in terms of technology. It results from both the high number of emission points and the specificity of the coking process, among others the high temperature and the reductive atmosphere prevailing in the coking chambers (Klejnowski et al. 2012; Sobolewski 2010). A workable and technologically justified method of reducing the mercury emission from the coking process involves removing mercury from coal before its use. However, the effectiveness of mercury removal from coal in the washing process is varied (Fig. 3), which should be related to the various modes of mercury occurrence in coal. Determining the mode of mercury occurrence in the inorganic constituents of coking coal will facilitate the selection of the most effective method of reducing mercury emission from coking plants.

### Identification of mercury occurrence in Polish coking coals

Within the framework of the conducted studies, the identification of the mode of mercury occurrence in inorganic constituents commonly occurring in Polish coking coal was performed. Taking into account the fact that mercury in coal is mainly associated with pyrite, special attention was devoted to sulfides. Furthermore, aluminosilicates, carbonates, sulfates, and oxides were analyzed.

**Fig. 4:**
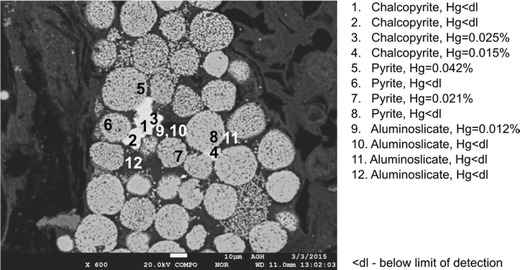
Results of mercury content determination in framboidal pyrite aggregates with chalcopyrite overgrowths (sample no. IV)

Figure 4 represents examples of the obtained results of the mercury content analysis in framboidal pyrite aggregates with chalcopyrite overgrowths surrounded by aluminosilicate phases. Pyrite and chalcopyrite as well as aluminosilicates contained significant amounts of mercury; however, higher contents of mercury, and also more frequently, were noticed for sulfides.

**Fig. 5:**
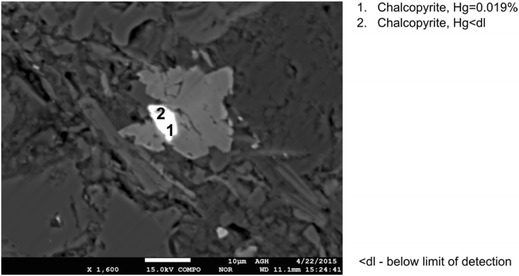
Results of mercury content determination in single chalcopyrite grain (sample no. III)

**Fig. 6:**
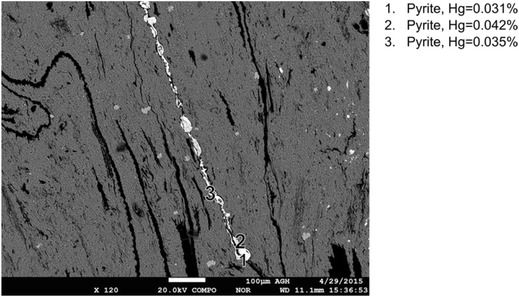
Results of mercury content determination in pyrite veins (sample no. I)

**Fig. 7:**
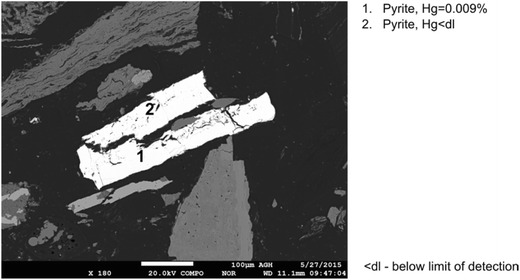
Results of mercury content determination in massive pyrite (sample no. VI)

**Fig. 8:**
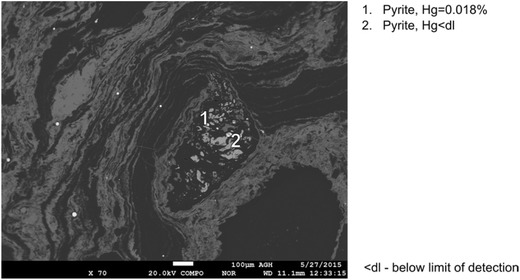
Results of mercury content determination in irregular pyrite grains (sample no. VII)

An inorganic constituent characterized by an especially high mercury content was chalcopyrite occurring both in overgrowths (Fig. 4) and as single grains (Fig. 5). A relatively high mercury content was also noticed for other forms of pyrite, i.e., veins filling cracks (Fig. 6), massive pyrite (Fig. 7), and irregular pyrite grains (Fig. 8).

**Fig. 9:**
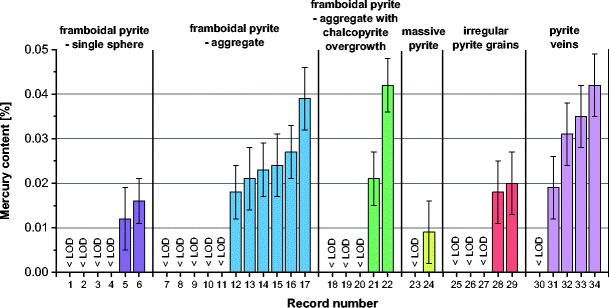
Comparison of mercury contents in various forms of pyrite (*< LOD* mercury content below the limit of detection)

**Fig. 10:**
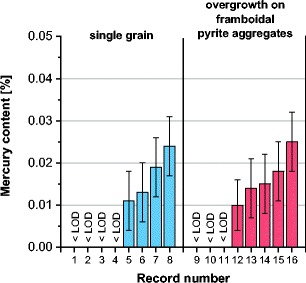
Comparison of mercury contents in various forms of chalcopyrite (*< LOD* mercury content below the limit of detection)

A comparison of mercury contents in various forms of pyrite and chalcopyrite recorded in the analyzed samples is given in Figs. 9 and 10. For framboidal pyrite, higher mercury contents were obtained for pyrites occurring in aggregates. In the case of other forms of pyrite, especially rich in mercury was the pyrite occurring in veins. Mercury contents in the analyzed pyrites, for the results above the limit of detection, were in the range from 0.009 to 0.042%. These values significantly exceeded the average mercury contents in the analyzed samples (55–313 μg/kg). Mercury contents in chalcopyrite remained at the same level in the range from 0.011 to 0.025%.

**Fig. 11:**
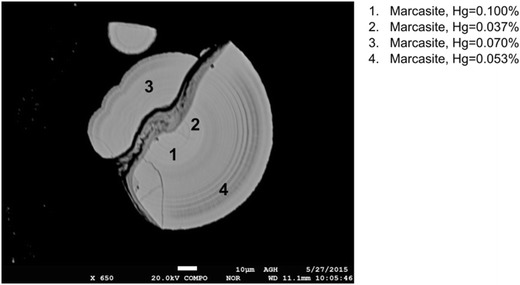
Results of mercury content determination in marcasite (sample no. VI)

Among all the analyzed sulfides, the highest mercury content was obtained for marcasite. Mercury contents in marcasite ranged from 0.037 to 0.100%. Despite a large number of analyses, only in the sample no. VI, one marcasite grain was noticed. However, it should be supposed that marcasite grains with very high mercury contents may occur in other coals as well. The analyzed marcasite grain is presented in Fig. 11.

Special attention needs to be paid to the recorded variable mercury content in single grains of the analyzed sulfides. This applies to pyrite and chalcopyrite as well as to marcasite. The observed variability of mercury content may be explained by the inclusion of other inorganic constituents, e.g., cinnabar (Diehl et al. 2004). It can also be supposed that in the analyzed sulfides, mercury can also occur in the form of sulfosalts which commonly occur with pyrite (Bolewski and Manecki 1993; Gaspar 2002).

In further research, aluminosilicates were examined. The examples of the obtained results are given in Fig. 4. Mercury contents above the detection limit were obtained in only 3 out of 19 performed analyses. It is worth mentioning that close to these aluminosilicates, mercury-rich grains of pyrite and chalcopyrite were noticed. Therefore, it can be supposed that mercury could diffuse from sulfides to these aluminosilicates. The diffusion of mercury from pyrite to other components of coal was noticed by Diehl et al. (2004). We also should not exclude the occurrence of microinclusions of sulfides with high mercury contents. It can be supposed that mercury was adsorbed by aluminosilicates.

**Fig. 12:**
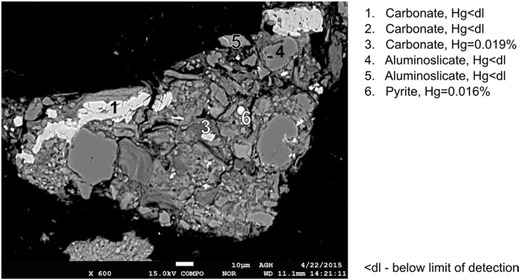
Results of mercury content determination in carbonates (sample no. IV)

Examples of the results of the carbonate analyses are shown in Fig. 12. In the case of 4 out of 14 performed microprobe analyses, mercury contents above the detection limit were obtained. The carbonate, for which a high mercury content was obtained each time, was iron carbonate (siderite).

**Fig. 13:**
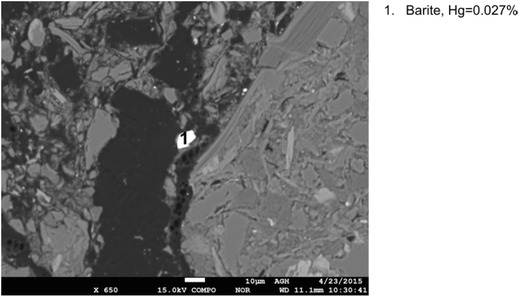
Results of mercury content determination in barite (sample no. III)

Mercury content above the limit of detection in barite (barium sulfate) was recorded. The analyzed grains are shown in Fig. 13. The obtained results confirm the possibility of occurrence of high mercury contents in heavy sulfates of hydrothermal origin. It should be mentioned that in the analyzed grains of ferric oxide, mercury occurrence was not observed.

**Fig. 14:**
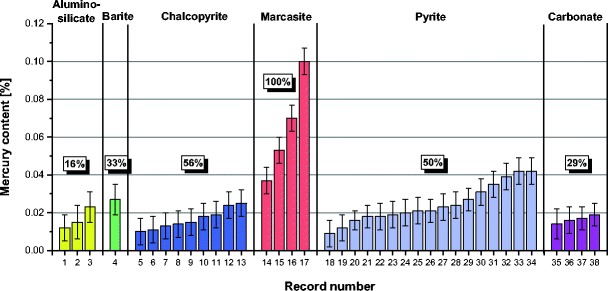
Comparison of mercury contents in analyzed inorganic constituents of coal (the percentage values represent frequencies of obtaining mercury content above the limit of detection for each inorganic constituent)

A comparison of the obtained results of mercury content measurements in the analyzed inorganic constituents of coal is shown in Fig. 14. Most frequently, mercury contents above the detection limit were obtained for sulfides and less often for carbonates and aluminosilicates. In the case of barite, because of a small number of the performed analyses, the results should not be generalized. Mercury content varied quite widely, also within each of the analyzed constituents. The highest mercury contents were obtained for marcasite in the range from 0.037 to 0.100%. High mercury contents were also noticed in pyrite (to 0.042%). The contents of mercury in chalcopyrite were observed below 0.025%, and those in the other analyzed inorganic constituents below 0.027%.

Based on the obtained results, mercury contents in the analyzed inorganic constituents cannot be clearly determined, because for a number of measurements, the contents of mercury were found to be below the limit of detection of the applied EPMA method, i.e., less than 0.006%. Nevertheless, a general conclusion can be formulated that significant amounts of mercury can occur in pyrite, chalcopyrite, marcasite, siderite, and barite as well as in parts of aluminosilicates in the vicinity of pyrite and chalcopyrite.

### Influence of mercury occurrence in coking coal on effectiveness of its removal

**Fig. 15:**
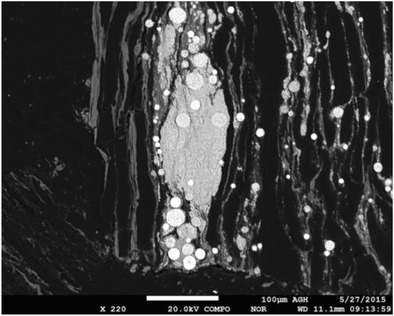
Overgrowths of pyrite on organic matter of coal (sample no. VI)

The studies showed both some similarities and a certain variety of the modes of mercury occurrence in the inorganic constituents of the examined coals. For each of the analyzed coal mines, very high contents of mercury in pyrite were noticed, but forms of pyrite were different for various coals (Table 4). A difference in the forms of pyrite could determine the effectiveness of its removal and the removal of mercury in the coal washing process (Diehl et al. 2004; Mastalerz and Drobniak 2005; Toole-O’Neil et al. 1999). The highest values of effectiveness of mercury removal were obtained in the case of coals for which the occurrence of the large framboidal pyrite aggregates with chalcopyrite overgrowths was noticed. The lowest amount of mercury was removed from coal for which only single grains of sulfides were found. In the case of the coal mine B, the occurrence of various forms of pyrite including large pyrite aggregates was noticed. This should result in a very high mercury removal effectiveness from coal. However, for the coal B, the occurrence of mercury-rich pyrites in middling products derived from the coal washing process was recorded. These pyrites often occurred as overgrowths on the organic matter of coal (Fig. 15), which could cause difficulties in their removal in the coal washing process. For that reason, the middling products were characterized by high mercury contents, which was also reported in other works (Kurus and Białecka 2016).

**Table 4:** Characteristics of sulfides occurring in analyzed samples

Coal mine	*η*_Hg_rejects_ [%]	Highest mercury content in sulfides [%]	Characteristics of sulfides
B	42	0.100	• Various forms of pyrites (veins, massive grains, irregular grains, large pyrite aggregates) • Marcasite
C	29	0.027	• Single grains of framboidal pyrites and chalcopyrites • Lack of large framboidal pyrite aggregates
E	84	0.042	• Large framboidal pyrite aggregates • Framboidal pyrite aggregates with chalcopyrite overgrowths
F	51	0.024	• Large framboidal pyrite aggregates

It could be supposed that carbonaceous overgrowths may occur also in other coals. Developing a method of removing this form of pyrite from hard coal may contribute to a significant reduction in mercury emission from the coking process as well as from other processes, i.e., coal combustion and coal gasification. It should be also mentioned that rejects from the coal washing process can be used in various sectors of the economy, among others, as a substitute for natural aggregates. In the case of mercury, the existing regulations define two issues: total mercury content and water-leachable mercury content. According to the results presented in Klojzy-Karczmarczyk et al. (2016), the rejects from the coal washing process meet both criteria.

## Conclusions

Based on the obtained results, it can be assumed that inorganic constituents which are removed from coal in the process of coal washing in the form of rejects may be characterized by very high mercury contents (up to 0.100%). Therefore, this process may allow for efficient mercury removal from coking coal and, thus, it will allow for reducing mercury emission from coking plants as well as for lowering mercury contents in coal coking products. The highest effectiveness of mercury removal was obtained in the case of coals for which the occurrence of the large framboidal pyrite aggregates with chalcopyrite overgrowths was noticed. The lowest amount of mercury was removed from coal for which only single grains of sulfides were found. The acquired knowledge of the modes of mercury occurrence in coal allows for the determination of the directions of further research as well as the directions of optimizing the technological parameters of the hard coal washing process.

The highest mercury contents obtained for these constituents were as follows: marcasite 0.100%, pyrite 0.042%, barite 0.027%, chalcopyrite 0.025%, aluminosilicates 0.023%, and carbonates (siderite) 0.019%. High contents of total mercury in the analyzed samples were related to the occurrence of mercury-rich inorganic constituents.

It has to be mentioned that Hg-rich sulfides were found in the middling products derived from the process of coking coal washing. These products are commonly used in the energy production sector. The high mercury contents in them may cause difficulties with their utilization in the near future.
